# The origin of motif families in food webs

**DOI:** 10.1038/s41598-017-15496-1

**Published:** 2017-11-23

**Authors:** Janis Klaise, Samuel Johnson

**Affiliations:** 1 Centre for Complexity Science, University of Warwick, Coventry, CV4 7AL United Kingdom; 2 School of Mathematics, University of Birmingham, Birmingham, B15 2TT United Kingdom

## Abstract

Food webs have been found to exhibit remarkable “motif profiles”, patterns in the relative prevalences of all possible three-species subgraphs, and this has been related to ecosystem properties such as stability and robustness. Analysing 46 food webs of various kinds, we find that most food webs fall into one of two distinct motif families. The separation between the families is well predicted by a global measure of hierarchical order in directed networks—trophic coherence. We find that trophic coherence is also a good predictor for the extent of omnivory, defined as the tendency of species to feed on multiple trophic levels. We compare our results to a network assembly model that admits tunable trophic coherence via a single free parameter. The model is able to generate food webs in either of the two families by varying this parameter, and correctly classifies almost all the food webs in our database. This is in contrast with the two most popular food web models, the generalized cascade and niche models, which can only generate food webs within a single motif family. Our findings suggest the importance of trophic coherence in modelling local preying patterns in food webs.

## Introduction

Food webs are abstract representations of which species consume which others in an ecosystem^1–3^. In a network-based description, species are represented by nodes and their trophic interactions are represented by directed links, pointing from prey to predator^2,4,5^. Much work has been devoted to understanding the origin and meaning of the particular trophic interaction patterns observed in these food webs^6–8^. Faced with the complexity of whole food webs, many researchers have focused on the interactions among subsets of species, through the analysis of small, connected subgraphs, or *motifs* ^9–13^.

The study of local interaction patterns via small network subgraphs^14^ first emerged in the study of neuronal and metabolic networks^15,16^. The methodology of analyzing the relative prevalence of small subgraphs with respect to a well-posed null model for network assembly remains the main way to gain an understanding of the local structural properties of networks, including food webs^11,12^.

**Figure 1:**
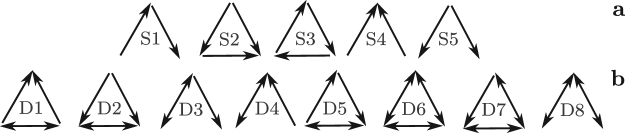
The 13 unique connected triads. These can be separated into two groups: (**a**) five triads, S1-S5, that only contain single links, (**b**) eight triads, D1-D8, that have double links (corresponding to mutual predation).

In this study we focus on the three-node connected triads of which there are 13 distinct ones (Fig. 1). Many of these triads admit a straightforward interpretation in the context of food-webs^12^. The eight triads D1–D8 have double links which correspond to mutual predation between two species. The five single link triads S1–S5 consist of some of the more basic building blocks of food webs. The triad S1 is a simple food chain^3,13^, S2 represents omnivory (a predator preying on two species at different trophic levels)^13,17^, triad S3 is a cycle (a relatively rare feature)^12,17^, and triads S4–S5 represent a predator preying on two species (apparent competition) and two predators sharing a prey species (direct competition), respectively^11^.

There are several competing hypotheses for the relative prevalence of these subgraphs in food webs. The prevailing hypotheses are that subgraphs emerge as a result of physical constraints (e.g species body size, abstracted by the niche dimension) in the assembly of food networks^9,12^, that functional importance leads to the observed structural patterns^18^, or that certain stability properties favour some subgraphs over others^11^.

Attempts to explain subgraph patterns using the two most established food web models, the generalized cascade model^19^ and the generalized niche model^20^, have been unsatisfactory since either model produces food webs with rigid three-species subgraph patterns^9,12^ while real food-webs display a far richer array of local preying patterns^11,12^. To remedy this disagreement between theory and observation, we study a new food-web model, the Generalized Preferential Preying Model (GPPM)^21^ which can accurately predict the three-species subgraph patterns across a wide array of distinct types of food-webs.

The ecological role of the omnivory triad S2 has been under particular scrutiny^9,11,12,22^. Different methodologies, however, have resulted in inconsistent claims about the prevalence of the omnivory triad in empirical food webs^9,11^ and its effect on food web stability is still unclear^22^. In this work we show that omnivory is a crucial feature that motivates a new classification of food webs which could provide insight into the controversy regarding the nature and role of omnivory^23^.

Local structural patterns in complex networks are intimately related to global network properties^24,25^. A network metric called trophic coherence was recently introduced in order to capture the degree to which the nodes fall neatly into distinct levels^21,26–28^. In the context of food webs, these are the trophic levels, and high coherence corresponds to the species at one level consuming almost exclusively species at the level immediately below (i.e. low omnivory). Trophic coherence was shown to be a major predictor of the linear stability of ecosystem models, as well as of a number of structural properties of empirical food webs^21^. It has also been related to the numbers of cycles in directed networks, and to the distribution of eigenvalues of associated matrices^26^.

Trophic coherence is a structural property of directed networks that places constraints on local topological features and on the prevalence of small subgraphs in particular. In this paper we present evidence that the relative prevalence of three-species subgraphs in food webs can be explained by the level of trophic coherence in both empirical and model food webs. This result provides another viewpoint in the debate about the origin of subgraph prevalences in food webs and further evidence of the importance of global organization in food webs^21^.

## Methods

### Quantifying triad significance

For any given network the exact number *N*_*k*_ of any of the *k* = 1, …, 13 connected three-node subgraphs (triads, Fig. 1) is influenced by the network size and the degree distribution of the vertices. To test the statistical significance of any given triad *k*, the empirically observed number *N*_*k*_ is compared against appearances of the same triad in a randomized ensemble of networks serving as a null model^16^. This comparison gives a statistical significance or *z*-score

1 $${z}_{k}=\frac{{N}_{k}-{\langle {N}_{k}\rangle }_{{\rm{rand}}}}{{\sigma }_{{\rm{rand}}}},$$

where ${\langle {N}_{k}\rangle }_{{\rm{rand}}}$ and ${\sigma }_{{\rm{rand}}}$ are the randomized ensemble average and standard deviation for triad *k*, respectively. The *z*-score of triad *k* thus measures the deviation of prevalence in the observed network with respect to the null model.

The *z*-scores of all 13 triads can be summarized in a triad significance profile (TSP) which is a vector ${\bf{z}}=\{{z}_{k}\}$ with components *z*_*k*_ for each triad *k*. Additionally, the normalized version of the TSP is often used to compare networks of different sizes and link densities^16^. This is given by

2 $$\hat{{\bf{z}}}=\{\frac{{z}_{k}}{\sqrt{\sum _{k=1}^{13}{z}_{k}^{2}}}\}.$$

The randomization procedure used to obtain the randomized ensemble statistics is a matter of choice. A careful selection of null model is important to discern between real effects and artefacts present in the TSP^29^. In our analysis, we follow the configuration model (CM) prescription^30,31^, and preserve the number of incoming and outgoing links for each node (the degree sequence) while randomizing links via a Markov chain Monte Carlo switching algorithm^15,16^. This preserves both the total number of nodes (species) and the links (trophic interactions) in the network. The generation of randomized networks and counts of triads was carried out with *mfinder*, the algorithm used by Milo *et al*. in their seminal work on network motifs^15,32^.

It is important to emphasize that the TSP is a relative measure of which triads are over- and under-represented with respect to the null model provided by the randomized CM networks. The over-(under-)representation as indicated by a positive (negative) *z*-score indicates that these triads appear more (less) frequently than in the randomized networks but do not imply an absolute saturation (absence) of said triads. Nevertheless, the TSP is an adequate tool for comparing networks of different sizes and degree distributions.

### Comparing networks based on triad significance

To quantitatively compare networks based on their TSP, we use Pearson’s correlation coefficient *r* between the normalized *z*-score vectors ${\hat{{\bf{z}}}}^{a}$ and ${\hat{{\bf{z}}}}^{b}$ of networks *a* and *b*, respectively^12,16^. This is defined as

3 $$r=\frac{\sum _{k=1}^{n}({\hat{z}}_{k}^{a}-{\bar{z}}^{a})({\hat{z}}_{k}^{b}-{\bar{z}}^{b})}{(n-1){\sigma }_{{\hat{{\bf{z}}}}^{a}}{\sigma }_{{\hat{{\bf{z}}}}^{b}}},$$

where

4 $${\bar{z}}^{a}=\frac{\sum _{k=1}^{n}{\hat{z}}_{k}^{a}}{n}$$

and

5 $${\sigma }_{{\hat{{\bf{z}}}}^{a}}=\sqrt{\frac{1}{n-1}\sum _{k=1}^{n}{({\hat{z}}_{k}^{a}-{\bar{z}}^{a})}^{2}}$$

are the mean and the standard deviation of the normalized *z*-score vectors, *a* and *b* specify the networks, *k* is an index over the triads and *n* = 13 is the total number of triads.

With this definition a value of *r* close to 1 indicates that the two networks have very similar TSPs and thus patterns of over- and under-represented triads, a value close to 0 indicates no similarity, and a value close to −1 indicates anti-similarity—i.e. triads over-represented in one network will typically be under-represented in the other (and vice versa).

Comparing the empirical networks is straightforward as we just calculate the *r*-coefficient pairwise for the *z*-score vectors of all 46 food webs in our database. On the other hand, for comparison with the model (described in a subsequent section), for each empirical network we fit our food-web model to the data, generate 1000 instances of a model network and then compute the *r*-coefficient of the empirical *z*-score vector and the average *z*-score vector of the model-generated ensemble.

### Clustering food webs into families

To uncover clusters of food webs with similar TSPs, we use a hierarchical, agglomerative clustering algorithm^33^ based on the Pearson’s correlation coefficient *r* between TSPs. First, we need to convert this to a distance measure. We define

6 $$d=\sqrt{\mathrm{2(1}-r)}.$$

This definition ensures that *d* is a Euclidean metric^34^ and we can readily apply hierarchical clustering. We use the UPGMA (average linkage) algorithm^33^ to uncover the full cluster hierarchy.

### Trophic coherence

Trophic coherence is a topological metric for directed networks that characterizes how layered the network is^21,26^. It measures the extent to which we can separate nodes into distinct groups so that any given group receives incoming links from just one other group and has outgoing links to another, different group of nodes. In the context of food webs, it measures the overall tendency of species to feed on multiple distinct trophic levels.

For each species *j* in the network, we define its *trophic level s*_*j*_ as the average trophic level of its prey, plus one^21,35^,

7 $${s}_{j}=1+\frac{1}{{k}_{j}^{{\rm{in}}}}\sum _{i}{a}_{ij}{s}_{i},$$

where ${k}_{j}^{{\rm{in}}}={\sum }_{i}{a}_{ij}$ is the number of prey of species *j* (also known as the *in-degree*) and *a*_*ij*_ are entries of the adjacency matrix *A* of the food web. Here the convention is that the directed trophic links point from prey *i* to predator *j*.

Because of the recursive nature of Eq. (7), to assign a trophic level to every node in the network two conditions must hold. First, there must be at least one node of zero in-degree – we call such nodes *basal*; and second, every node in the network must be reachable by a path from at least one basal node. Food webs satisfy both conditions so the linear system defined by Eq. (7) has a unique solution. Without loss of generality we assign *s*_*j*_ = 1 for all basal species, as is the convention in ecology.

We define the *trophic distance* associated to link *a*_*ij*_ in the network as the difference between the trophic levels at the endpoints, ${x}_{ij}={s}_{j}-{s}_{i}$. Note that this is not a distance in the mathematical sense as it can take negative values. Denote by *p*(*x*) the distribution of trophic distances as measured on a network. This will have mean 〈*x*〉 = 1 by definition and a standard deviation $q=\sqrt{\langle {x}^{2}\rangle -1}$ which we will call the *trophic incoherence* parameter.

**Figure 2:**
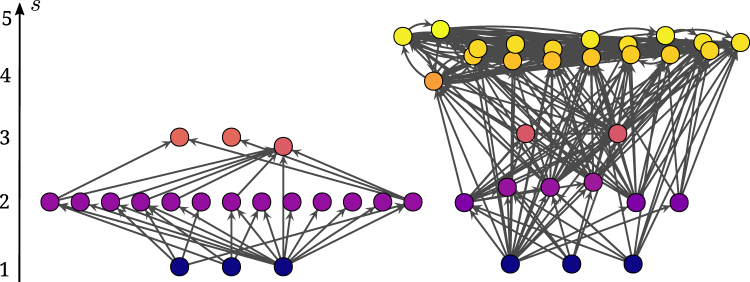
Examples of different degrees of trophic coherence in food webs. Left: Crystal Lake (Delta)—a highly coherent network with *q* = 0.17, note that only one node prevents the network from being perfectly coherent (*q* = 0). Right: Coachella Valley—an incoherent network with *q* = 1.21, note the high number of nodes falling between integer trophic levels due to the complex patterns of trophic links.

The trophic incoherence parameter is thus a measure of the homogeneity of the distribution *p*(*x*). For perfectly coherent networks we have *q* = 0, which translates to having only integer valued trophic levels and all species feeding on prey only one trophic level below their own. In this case the network is perfectly structured, or layered, as there are distinct groups of herbivores feeding only on basal species, predators feeding only on herbivores and so on. For less coherent networks, *q* > 0 indicates a less ordered trophic structure, where trophic levels take fractional values and species tend to prey on a broader range of trophic levels. See Fig. 2 for examples of coherent and incoherent food webs.

### Model with tunable trophic coherence

Various mathematical models of food webs have been proposed to capture and explain different aspects of food webs^19,20,36–38^, but the main models still fail to capture the full variety of empirically observed structures^12,21,39,40^. To reproduce many of the empirical structures^21^, in particular the prevalence of three-species motifs, we propose a model for food webs that allows us to adjust the incoherence parameter *q* by means of fitting a single free parameter. The model is a generalization of the Preferential Preying Model (PPM) introduced in ref.^21^, with the improvement that it can generate bidirectional links and cycles of higher order, thus producing more realistic networks. In the following we denote by *B*, *N* and *L* the number of basal nodes, total nodes and links in the network respectively, all parameters to be fitted using the empirical network data.

We begin with *B* basal nodes and no links. We assign trophic levels *s* = 1 to all basal nodes. We then add *N* − *B* new nodes to the network sequentially according to the following rule. For each new node *j*, pick exactly one prey *i* at random from among all the existing nodes in the network, thus creating a link from *i* to *j*. In doing so, we define the temporary trophic level of node *j* as ${\hat{s}}_{j}=1+{\hat{s}}_{i}$. After this procedure finishes, we have a network of *N* nodes and *N*−*B* links, and each node has a (temporary) trophic level ${\hat{s}}_{i}$.

Once all *N* nodes are created, we add the remaining links to the network to bring the expected number of links up to *L*. The links are chosen among all possible pairs of nodes (*i*, *j*) where *j* is not a basal node (this ensures no incoming links to basal nodes which would make them non-basal), with a probability *P*_*ij*_ that decays with the (temporary) trophic distance ${\hat{x}}_{ij}={\hat{s}}_{j}-{\hat{s}}_{i}$ between them. Specifically, we set

8 $${P}_{ij}\propto \exp (-\frac{{({\hat{x}}_{ij}-1)}^{2}}{2{T}^{2}}),$$

where *T* is a free parameter which sets the degree of prey diversity between multiple trophic levels. This form of probability ensures that the most likely links to be created are between adjacent (temporary) trophic levels. The probabilities in Eq. (8) are normalized so that the expected number of links in the final network is *L*.

At the end of the network creation procedure the trophic levels need to be recalculated according to Eq. (7) as the addition of new links will have changed the network topology, and the trophic levels in the final network need not correspond to the temporary integer valued trophic levels.

**Figure 3:**
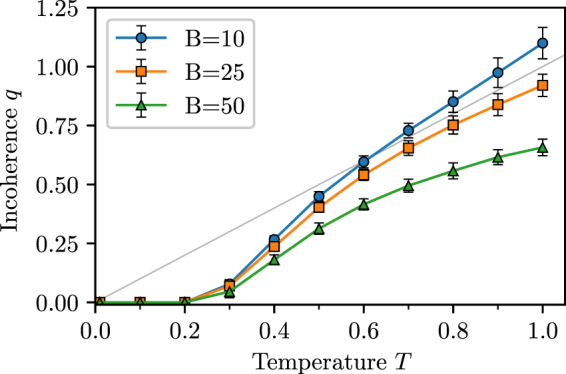
Dependence of the incoherence parameter *q* on the temperature parameter *T*. Simulated ensembles of networks have *N* = 100 nodes *B* of which are basal and average non-basal degree $\langle k\rangle =L/(N-B)=10$. The averages are computed over at least 1000 networks and error bars are one standard deviation of the sample. The grey line is $f(x)=x.$

The free parameter *T* is analogous to temperature in statistical physics and sets the amount of deviation from a perfectly coherent network. For *T* = 0, only links between adjacent (temporary) trophic levels are allowed which results in the incoherence parameter *q* = 0. In this case the temporary trophic levels coincide with the actual trophic levels as the addition of links does not change the initially assigned trophic levels. As *T* is increased, links between a wider range of (temporary) trophic levels become more probable, so we expect *q* > 0 and increasingly more random networks. A sample dependence of *q* on *T* is shown in Fig. 3. The model exhibits a monotonic dependence of the incoherence parameter *q* on temperature *T* which provides a basis for fitting the model to empirical food webs given the empirically observed *q*. We also find that the level of incoherence that is achieved at any given temperature depends on *B*/*N*, the ratio of basal species to all species. We will further explore this relationship in the subsequent section.

To fit the model to the food web data, we provide as input the number of basal species *B*, the number of total species *N*, and the number of links or trophic interactions *L*. We then use stochastic root finding to find the value of the temperature parameter *T* that results in an ensemble of networks whose incoherence parameter *q* is centred about the empirical incoherence parameter as measured from the food web topology.

### Empirical food web data

We study the triad significance profile (TSP) in 46 empirical food webs from a variety of environments: marine, freshwater (river and lake) and terrestrial. Table 1 gives the relevant summary statistics of each food web. The full structure of each food web is included in supplementary information.

**Table 1:** An alphabetical list of the 46 food webs studied in the paper.

Food web	*N*	*B*	*L*	*q*	*T*	Type	Reference	ID
Akatore Stream	84	43	227	0.16	0.40	River	41–43	5
Benguela Current	29	2	196	0.69	0.65	Marine	44	39
Berwick Stream	77	35	240	0.18	0.40	River	41–43	7
Blackrock Stream	86	49	375	0.19	0.42	River	41–43	9
Bridge Broom Lake	25	8	104	0.53	0.64	Lake	45	28
Broad Stream	94	53	564	0.14	0.37	River	41–43	1
Canton Creek	102	54	696	0.15	0.38	River	43	4
Caribbean (2005)	249	5	3302	0.73	0.69	Marine	46	41
Caribbean Reef	50	3	535	0.94	0.82	Marine	47	43
Carpinteria Salt Marsh Reserve	126	50	541	0.65	0.85	Marine	48	36
Caitlins Stream	48	14	110	0.20	0.41	River	41–43	12
Chesapeake Bay	31	5	67	0.45	0.62	Marine	49,50	26
Coachella Valley	29	3	243	1.21	1.02	Terrestrial	51	45
Coweeta (1)	58	28	126	0.30	0.52	River	41–43	20
Crystal Lake (Delta)	19	3	30	0.17	0.43	Lake	52	6
Cypress (Wet Season)	64	12	439	0.63	0.66	Terrestrial	53	34
Dempsters Stream (Autumn)	83	46	414	0.21	0.43	River	41–43	13
El Verde Rainforest	155	28	1507	1.01	0.99	Terrestrial	54	44
Everglades Graminoid Marshes	64	4	681	1.35	1.10	Terrestrial	55	46
Florida Bay	121	14	1767	0.59	0.59	Marine	53	29
German Stream	84	48	352	0.20	0.43	River	41–43	11
Grassland (U.K.)	61	8	97	0.40	0.69	River	56	24
Healy Stream	96	47	634	0.22	0.42	River	41–43	15
Kyeburn Stream	98	58	629	0.18	0.41	River	41–43	8
LilKyeburn Stream	78	42	375	0.23	0.44	River	41–43	18
Little Rock Lake	92	12	984	0.67	0.65	Lake	57	37
Lough Hyne	349	49	5102	0.60	0.60	Lake	58,59	31
Mangrove Estuary (Wet Season)	90	6	1151	0.67	0.63	Marine	53	38
Martins Stream	105	48	343	0.32	0.51	River	41–43	21
Maspalomas Pond	18	8	24	0.49	1.01	Lake	60	27
Michigan Lake	33	5	127	0.37	0.48	Lake	61	22
Narragansett Bay	31	5	111	0.61	0.68	Marine	62	33
Narrowdale Stream	71	28	154	0.23	0.44	River	41–43	17
N.E. Shelf	79	2	1378	0.73	0.66	Marine	63	42
North Col Stream	78	25	241	0.28	0.45	River	41–43	19
Powder Stream	78	32	268	0.22	0.42	River	41–43	14
Scotch Broom	85	1	219	0.40	0.54	Terrestrial	64	23
Skipwith Pond	25	1	189	0.61	0.54	Lake	65	32
St. Marks Estuary	48	6	218	0.63	0.67	Marine	66	35
St. Martin Island	42	6	205	0.59	0.63	Terrestrial	67	30
Stony Stream	109	61	827	0.15	0.38	River	43	3
Sutton Stream (Autumn)	80	49	335	0.15	0.40	River	41–43	2
Troy Stream	77	40	181	0.19	0.42	River	41–43	10
Venlaw Stream	66	30	187	0.23	0.44	River	41–43	16
Weddell Sea	483	61	15317	0.72	0.68	Marine	68	40
Ythan Estuary	82	5	391	0.42	0.50	Marine	69	25

From left to right, the columns are for: name, number of species *N*, number of basal species *B*, number of links *L*, ecosystem type, trophic incoherence parameter *q*, value of the temperature parameter *T* found to yield (on average) the empirical *q* with our model, references to original work, and the numerical ID.

## Results

### Motifs in empirical food webs

**Figure 4:**
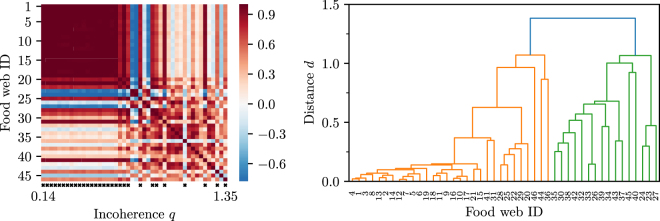
Pearson’s correlation coefficient of the triad significance profiles (left) and clustering of food webs into two families (right). Left: The coefficient is measured pairwise between all pairs of empirical food webs. Warmer colours indicate greater similarity while colder colours indicate dissimilarity. The food webs are arranged according to increasing incoherence parameter (left to right and top to bottom). Black crosses just below the heatmap indicate membership to Family 1 according to a clustering algorithm. Right: Dendrogram of the hierarchical clustering algorithm applied to food webs based on the distance $d=\sqrt{\mathrm{2(1}-r)}.$ A threshold distance ${d}_{c}=1.1$ uncovers two large families with smaller subclusters within.

**Figure 5:**
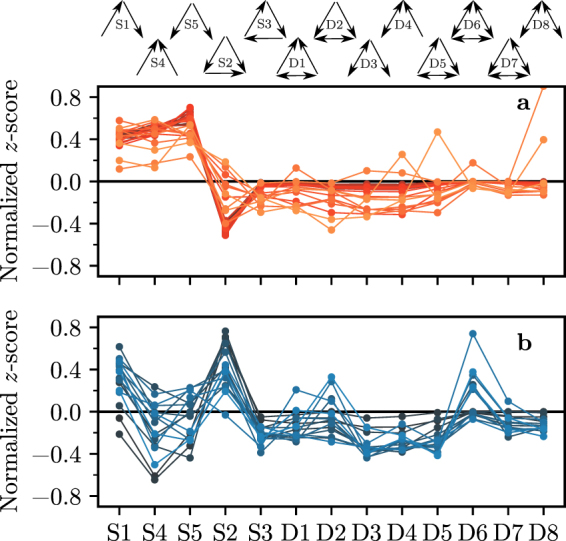
Triad significance profiles (TSP) as measured by the normalized *z*-score of the two groups of food webs. (**a**) Food webs in the first family (ID 1–22,25,28,29,31,36,41,44,46) with low incoherence parameter *q* characterized by an over-representation of triads S1, S4 and S5 and an under-representation of triad S2. (**b**) Food webs in the second family (ID 23,24,26,27,30,32–35,37–40,42,43,45) with high incoherence parameter characterized by an over-representation of triad S2.

The main results are summarized in Figs 4 and 5.

Figure 4 shows the pairwise Pearson correlation coefficients of the triad significance profiles between all 46 food webs. The food webs are arranged by increasing incoherence parameter *q* so that more coherent food webs are assigned a lower ID. Red hue or warmer colours indicate a larger coefficient, while blue hue or colder colours indicate an anti-correlation in the TSPs.

We see that roughly two families of food webs emerge with similar TSPs. The first family (roughly ID 1-22) is characterized by relatively high coherence (low incoherence parameter *q*), for which the similarities in the TSPs are very high ($r\ge 0.8$).

There is a second family of food webs, characterized by a high incoherence parameter *q*, that also show high similarities in their TSPs. Membership to this second family is not as clear as there is a tighter core of food webs belonging to it, with a periphery that only shares some similarities.

To make these ideas more precise, we performed hierarchical clustering of food webs based on a distance metric derived from the pairwise Pearson correlation coefficients. The resulting clusters are shown as a dendrogram in Fig. 4. By choosing a threshold distance *d*_*c*_, we can group food webs into a number of distinct families based on the similarities of their TSPs. Setting ${d}_{c}=1.1$, we identify two families which include all webs. Family 1 consists of food webs with ID 1–22, 25, 28, 29, 31, 36, 41, 44, 46 whereas Family 2 contains webs with ID 23, 24, 26, 27, 30, 32–35, 37–40, 42, 43, 45. We also observe that these larger families contain within themselves smaller, even more closely related clusters (e.g ID 1–22 corresponding to very low *q*).

Setting a lower threshold distance could provide a more fine-grained classification of food webs in more than two distinct families but we now show that this coarse classification into two families allows us to qualitatively differentiate food webs based on species preying patterns, specifically the extent of omnivory. To this end, we look closer at the bulk behaviour of the TSPs for the two families. Figure 5 shows the normalized profiles of Family 1 (top) and Family 2 (bottom).

We first consider Family 1. The bulk behaviour of food webs in this family is characterized by an over-representation of triads S1, S4 and S5, as well as an under-representation of triad S2 (with the exception of ID 22 Michigan Lake, ID 29 Florida Bay and ID 46 Everglades Graminoid Marshes). We should find the pattern of under-representation of triad S2 (which represents omnivory) unsurprising, since the majority of food webs belonging to this family have a low incoherence parameter *q*, which limits the ability of species to feed on multiple different trophic levels. Equally, the over-representation of triads S1, S4 and S5 is to be expected as these are the only three triads out of 13 that can arise in a hypothetical food web with *q* = 0, which is a value close to the empirical values of *q* for food webs in this family. The double link triads D1-D8 are all under-represented or close to even, in agreement with our expectations.

We now turn to Family 2. Here the triads S1, S4 and S5 no longer follow a strong pattern of over-representation and the double link triads D1-D8 are not always under-represented. The most distinguishing feature, however, is the bulk over-representation of triad S2 (with the exception of ID 40 Weddell Sea), in stark contrast to Family 1. We will argue that this is the main feature that separates the two food web families.

This pattern of food webs based on the under- or over-representation of triad S2 was alluded to in previous work^12^, however it is in disagreement with the predictions of the generalized cascade^19^ and niche^20^ models which can only produce food webs where S2 is over-represented^12^. Subsequently, we present results from our model which show that it is possible to change the pattern of under-representation to over-representation of triad S2 by increasing the incoherence parameter *q*, thus providing evidence that trophic coherence can naturally give rise to two food web families characterized by low or high prevalence of omnivory, respectively.

### Comparison between empirical and model networks

**Figure 6:**
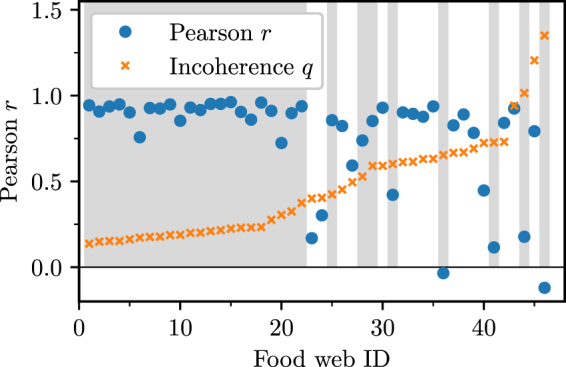
Pearson’s correlation coefficient of the triad significance profiles (TSP). The coefficient is measured between the empirical TSP and the average TSP in the model ensemble over 1000 simulated networks. Food webs are arranged by increasing incoherence parameter *q*. The grey shading indicates membership to the first family.

We have also investigated the similarities of triad significance profiles between the empirical food webs and model generated food webs. To this end we study the similarity of the TSPs between each empirical food web and an ensemble of model food webs fitted to the data of the empirical one. The results are summarized in Fig. 6. Averaging over an ensemble of 1000 model generated food webs fitted to each empirical food web, we measured the Pearson correlation coefficient between the TSP of the empirical food web and the TSP of the ensemble average. The results show that the model is able to reproduce empirically observed TSPs for the majority of food webs in both families with high accuracy. The model fails to produce accurate TSPs for a number of food webs and sometimes even produces anti-correlated TSPs (*r* < 0). If we require that *r* > 0.5, eight food webs are not able to be reproduced accurately by our model, five in Family 1 (ID 31 Lough Hyne, ID 36 Carpinteria Salt Marsh Reserve, ID 41 Caribbean Reef, ID 44 El Verde Rainforest and ID 46 Everglades Graminoid Marshes) and three in Family 2 (ID 23 Bridge Broom Lake, ID 24 Grassland (U.K.) and ID 40 Weddell Sea). Recall that IDs are assigned in the order of increasing *q* so these particular food webs are unusual members of their respective families in that they tend to have extreme values of *q* with respect to the majority of networks in either family (higher than average in Family 1 and lower than average in Family 2). Because of the imperfect agreement between *q* and family membership, our model cannot replicate the structure of these sporadic webs. This suggests that for some food webs information about trophic coherence *q* may not be enough to reproduce realistic looking TSPs and there may be further mechanisms of prey selection at play^12^.

### The role of omnivory and basal species

We now focus on the claim that the main difference between the two families of food webs is the relative under- and over-representation of triad S2, or the degree of omnivory in a food web. A prevalence of triad S2 indicates that the species in a food web often feed on different trophic levels, contributing to an increased incoherence parameter *q* as discussed at the start of this section. A scarcity of triad S2, on the other hand, indicates that species only tend to feed on prey with similar trophic levels, which in turn signals a low incoherence parameter. This suggests a relationship between the *z*-score of triad S2 and network incoherence as measured by *q*.

Furthermore, model results (Fig. 3) suggest that a high proportion of basal species to all species, *B*/*N*, produces more coherent food webs (i.e. with a low incoherence parameter *q*). We take this as an additional predictive food web statistic for family membership.

**Figure 7:**
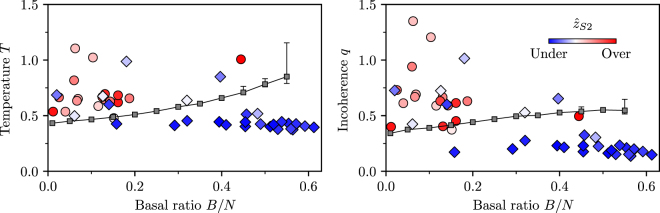
Scatter plots of the temperature *T* (left) and the incoherence parameter *q* (right) versus the basal species ratio *B*/*N* for all food webs. The gradient indicates the degree of over-representation (red circles) or under-representation (blue diamonds) of the feed-forward triad S2 as measured by the normalized z-score ${\hat{z}}_{{\rm{S2}}}$. The line shows the transition from over-representation (above) to under-representation (below) as observed in the model with *N* = 100, $\langle k\rangle =L/(N-B)=10$ averaged over 100 runs. Error bars are approximate 95% confidence intervals.

Our findings are summarized in Fig. 7. This is a scatter plot of all 46 food webs where we have plotted the fitted model temperature *T* and the measured incoherence parameter *q* against the ratio of basal species to all species *B*/*N*. We observe a clear anti-correlation between *q* and *B*/*N* (linear model $q=a\tfrac{B}{N}+b$: $a=-1.06$, $b=0.77$, ${R}^{2}=0.53$, $p=8.47\cdot {10}^{-9}$) that indicates a positive relationship between how coherent a network is (low *q*) and how many of its species are basal.

We have also coloured the markers of each food web to indicate the level of over- or under-representation of triad S2 as measured by the normalized *z*-score ${\hat{z}}_{{\rm{S2}}}$. Red circles indicate an over-representation while blue diamonds indicate an under-representation of S2 in the respective food web. Remarkably, based on this measure, we uncover two clusters of food webs corresponding roughly to the two families based on TSP similarities. The first cluster is once again characterized by a high incoherence parameter *q* as well as a low ratio of basal species to all species *B*/*N*. The second cluster is characterized by a low incoherence parameter and a high ratio of basal species to all species. The only exceptions are six food webs in the first family (ID 20 Coweeta (1), ID 21 Martins Stream, ID 31 Lough Hyne, ID 36 Carpinteria Salt Marsh Reserve, ID 41 Caribbean Reef and ID 44 El Verde Rainforest), four of which correspond to food webs poorly matched by our model (Fig. 6). We conclude that, indeed, the main difference between the two families is the relative role of triad S2 as already observed in the bulk behaviour of the TSPs in Fig. 5.

Finally, we study whether our model exhibits a similar transition from a relatively S2-poor to an S2-rich state which would explain the relatively good agreement between empirical and model generated TSPs for the two families (Fig. 6). We find that for a given basal species ratio *B*/*N* there exists a critical temperature ${T}_{c}$, and thus a critical incoherence parameter *q*_*c*_, which signifies such a transition. For *T* (and *q*) below these critical values, the model generates networks where S2 is under-represented, while for values above critical, the networks generated have either an even or an over-represented number of S2 triads. We include the transition line of the two regimes in Fig. 7 for an ensemble of 100 model networks with *N* = 100 species and an average (non-basal) degree $\langle k\rangle =L/(N-B)=10$. Networks with *q* below the line show an under-representation of S2 triads, while networks with *q* above the line show an over-representation as measured by ${\hat{z}}_{{\rm{S2}}}$.

Remarkably, the model results are in very good agreement with the empirical data despite the fact that both the network size *N* and the average degree *k* vary considerably between the empirical food webs. Almost all food webs with an under-represented number of S2 triads fall below the transition line of the model while those with an over-represented number reside above the line.

These findings suggest that the two families of food webs differ in the degree of omnivory present as measured by the prevalence of triad S2 which is itself intimately related to the incoherence parameter *q*. Interestingly, based on the strong anti-correlation between *q* and *B*/*N*, either parameter is a strong determinant of family membership. To our knowledge, the GPPM is the first food-web model able to reproduce triad significance profiles consistent with empirical observations. The ability to produce model networks belonging to either of the two families suggests that the parameters *q* and *B*/*N* are both important in the mathematical modelling of food webs and may, in fact, be fundamental for understanding local preying patterns in food webs.

## Discussion

Our investigation of trophic interaction patterns in food webs has revealed significant correlations between the degree of omnivory, hierarchical organization of trophic species and the density of basal species.

The analysis of local trophic interactions via triad significance profiles in empirical food webs reveals two distinct families of food webs characterized by a relatively low or high incoherence parameter respectively. While certain differences across families of food webs based on their TSPs have been observed before^12^, these are not predicted by any existing food web models, calling into question their use as null models given the academic significance attached to food web motifs^9–13^. Trophic coherence provides a network theoretic metric that enables us to classify and predict the relative prevalence of such motifs.

We have shown qualitatively that the the main difference between the two food web families is the extent of omnivory, as measured by the over- or under-representation of triad S2 (the feed-forward loop). This classification of food webs into two families according to the extent of omnivory is at odds with previous claims that omnivory occurs more often than one would expect to happen by chance across most food webs^12^. On the other hand, the existence of these families may be related to different ways omnivory emerges in food webs and influences their stability^21–23^. We have tested our prediction for the onset of omnivory using a new model for generating synthetic food webs with a given trophic coherence. We find that the model exhibits a transition from an under-representation of omnivory to an over-representation of omnivory as a function of trophic coherence. Our model results fit the food web data very well, providing evidence of the importance of trophic coherence as well as the basal species density in modelling realistic trophic interactions. We would like to emphasize that these findings are remarkably robust between food webs originating from vastly different habitats.

This work has expanded on the importance of trophic coherence in predicting structural features in food webs^21^, but the biological origin of trophic coherence remains elusive. Basal species density and its effect of suppressing highly incoherent structures in both empirical and model food webs may provide some clues. All other things being equal, a higher proportion of autotrophs in a food web will necessarily mean that a higher proportion of consumers will feed on these basal species. In turn, this would have a dampening effect on the formation of long food chains in the trophic hierarchy and hence fewer possibilities for a varied diet of species at the top. Figure 2 exemplifies how this hypothesis could lead to very different food web structures. Established food web models do not treat basal species density as a predictor for emergent structure but rather as an emergent property itself. On the other hand, most food webs have been found to be significantly more trophically coherent than a random graph with the same density of basal species, so there must be other coherence-inducing mechanisms at play^26^. Further work is needed to elucidate the reasons behind this property of ecosystem structure.

## Electronic supplementary material

Supplementary Information

Supplementary Dataset 1

Supplementary Dataset 2

Supplementary Dataset 3

Supplementary Dataset 4

Supplementary Dataset 5

Supplementary Dataset 6

Supplementary Dataset 7

Supplementary Dataset 8

Supplementary Dataset 9

Supplementary Dataset 10

Supplementary Dataset 11

Supplementary Dataset 12

Supplementary Dataset 13

Supplementary Dataset 14

Supplementary Dataset 15

Supplementary Dataset 16

Supplementary Dataset 17

Supplementary Dataset 18

Supplementary Dataset 19

Supplementary Dataset 20

Supplementary Dataset 21

Supplementary Dataset 22

Supplementary Dataset 23

Supplementary Dataset 24

Supplementary Dataset 25

Supplementary Dataset 26

Supplementary Dataset 27

Supplementary Dataset 28

Supplementary Dataset 29

Supplementary Dataset 30

Supplementary Dataset 31

Supplementary Dataset 32

Supplementary Dataset 33

Supplementary Dataset 34

Supplementary Dataset 35

Supplementary Dataset 36

Supplementary Dataset 37

Supplementary Dataset 38

Supplementary Dataset 39

Supplementary Dataset 40

Supplementary Dataset 41

Supplementary Dataset 42

Supplementary Dataset 43

Supplementary Dataset 44

Supplementary Dataset 45

Supplementary Dataset 46
