# Supplementary material for: The origin of motif families in food webs
Source: Sci Rep. 2017 Nov 23;7:16197. doi: 10.1038/s41598-017-15496-1 (PMC5700930; doi:10.1038/s41598-017-15496-1)
Supplement: Supplementary file 1 — Supplementary Information [file 41598_2017_15496_MOESM1_ESM.pdf]

Supplementary Information for *The origin of motif families in food webs*

Janis Klaise Samuel Johnson

Uncentred Pearson's correlation coefficient

An alternative to the usual Pearson's correlation coefficient used to compare triad significance profiles in the main text (Equation 3) is to use the uncentred Pearson's correlation coefficient[1] defined as

$$r^u = \frac{\sum_{k=1}^{n} \hat{z}_k^a \hat{z}_k^b}{\sigma_{\hat{\mathbf{z}}^a} \sigma_{\hat{\mathbf{z}}^a}}. \tag{1}$$

We performed our analysis of comparisons between food web TSPs using this alternative measure and our findings are summarized in Figures 1 and 2. We note that the results are largely unchanged when using this alternative similarity metric.

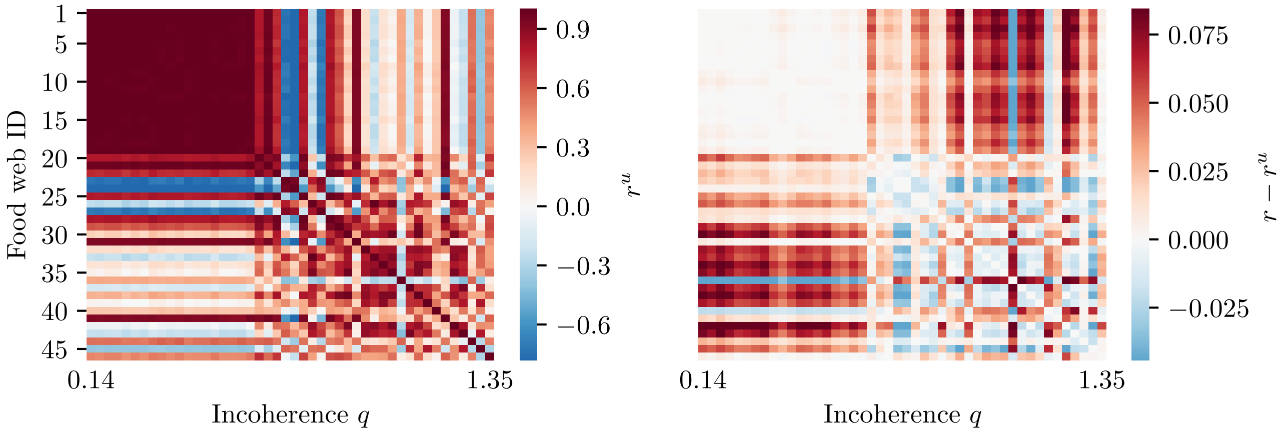

Figure 1: **Uncentred Pearson's correlation coefficient of the triad significance profiles (left) and the difference between Pearson's r and uncentred r^u (right).** We note that the results are qualitatively identical to results obtained using the usual Pearson's r (left). The difference between the two metrics is an order of magnitude smaller than the measured correlations (right).

References

[1] Stouffer, D. B., Camacho, J., Jiang, W. & Amaral, L. A. N. Evidence for the existence of a robust pattern of prey selection in food webs. *Proceedings of the Royal Society of London B: Biological Sciences* **274**, 1931–1940; DOI: 10.1098/rspb.2007.0571 (2007).

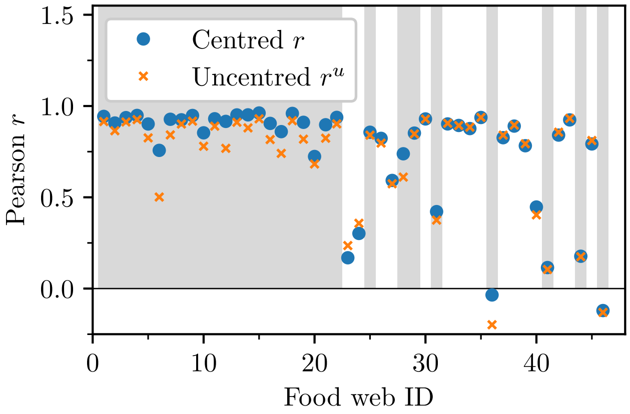

Figure 2: **Comparison of the predictive power of the centred and the uncentred Pearson's r.** We do not observe major discrepancies between results using the uncentred Pearson's r^u over the usual Pearson's r.
